# Protective Mechanism of Leucine and Isoleucine against H_2_O_2_-Induced Oxidative Damage in Bovine Mammary Epithelial Cells

**DOI:** 10.1155/2022/4013575

**Published:** 2022-03-22

**Authors:** Sainan Wu, Xize Liu, Long Cheng, Duojia Wang, Guixin Qin, Xuefeng Zhang, Yuguo Zhen, Tao Wang, Zhe Sun

**Affiliations:** ^1^College of Animal Science and Technology, JLAU-Borui Dairy Science and Technology R&D Center, Key Laboratory of Animal Nutrition and Feed Science of Jilin Province, Key Laboratory of Animal Production Product Quality and Security Ministry of Education, Jilin Agricultural University, Changchun 130118, China; ^2^Institute of Animal Science, Jilin Academy of Agricultural Sciences, Changchun 130033, China; ^3^Postdoctoral Scientific Research Workstation, Feed Engineering Technology Research Center of Jilin Province, Changchun Borui Science & Technology Co., Ltd., Changchun 130114, China; ^4^College of Life Science, Jilin Agricultural University, Changchun 130118, China

## Abstract

Leucine and isoleucine possess antioxidative and anti-inflammatory properties. However, their underlying protective mechanisms against oxidative damage remain unknown. Therefore, in this study, the protective mechanism of leucine and isoleucine against H_2_O_2_-induced oxidative damage in a bovine mammary epithelial cell lines (MAC-T cells) were investigated. Briefly, MAC-T cells exposed or free to H_2_O_2_ were incubated with different combinations of leucine and isoleucine. The cellular relative proliferation rate and viability, oxidative stress indicators, and inflammatory factors were determined by specific commercial kits. The genes related to barrier functions was measured by real-time quantitative PCR. The protein expression differences were explored by 4D label-free quantitative proteomic analyses and validated by parallel reaction monitoring. The results revealed that leucine and isoleucine increased cell proliferation, total antioxidant status (TAS), and the relative mRNA expression of occludin, as well as decreased malondialdehyde (MDA), total oxidant status (TOS)/TAS, IL-6, IL-1*β*, and TOS. When leucine and isoleucine were combined, MDA, TOS/TAS, and the relative mRNA expression levels of claudin-1, occludin, and zonula occludens-1 increased when compared to leucine or isoleucine alone. Proteomics analyses revealed that leucine significantly upregulated the propanoate metabolism; valine, leucine, and isoleucine degradation; and thermogenesis pathways, whereas isoleucine significantly upregulated the peroxisome and propanoate metabolism pathways. In conclusion, leucine protected MAC-T cells from H_2_O_2_-induced oxidative stress by generating more ATP to supplement energy demands, and isoleucine improved the deficit in peroxisome transport and promoted acetyl-CoA production. The findings of this study enhance our understanding of the protective mechanisms of leucine and isoleucine against oxidative damage.

## 1. Introduction

The transition period from pregnancy to lactation is critical for dairy cow health, production, and profitability, because alternations in energy metabolism can negatively affect their health, including conditions such as mastitis and retained placenta [1]. The considerable increase in oxygen requirements due to increased metabolic demands results in the augmented production of reactive oxygen species (ROS) [2]. Oxidative stress is primarily caused by an imbalance between free radical formation and elimination, that is, increased ROS production and/or reduced antioxidant defense, which leads to lipid peroxidation, exacerbated inflammatory responses, and cell damage [3–5]. If mammary glands are subjected to oxidative stress, the blood-milk barrier is destroyed, thereby resulting in lower milk yield and quality [6]. Therefore, it is a necessary and urgent matter to identify effective antioxidants that relieve the oxidative stress in the mammary glands. MAC-T cells, a bovine mammary epithelial cell line widely used as a model for lactation researches, were utilized in this study [7–9].

Amino acids, especially branched-chain amino acids (BCAAs), play important roles in cellular metabolism and stress responses [10–13]. BCAAs including leucine, isoleucine, and valine are proteinogenic essential amino acids with aliphatic-branched side chains [14]. In the serum metabolites of early lactating dairy cows, a significant association between BCAA concentrations and oxidative stress indicators was observed [15]. Mao et al. [16] suggested that the supplementation of leucine in early weaned Hu lambs was beneficial to their health as indicated by the increased total antioxidant capacity (T-AOC) and glutathione peroxidase (GSH-PX) level and by decreased H_2_O_2_ concentration in the plasma. Yin et al. [17] found that dietary isoleucine supplementation in hybrid bagrid catfish reduced ROS and malondialdehyde (MDA) levels and upregulated tight junction structure, which thereby antagonized oxidative damage and physical barrier functions. However, the underlying protective mechanisms of leucine and isoleucine against oxidative damage in MAC-T cells remain unknown.

Therefore, in this study, the protective mechanism of leucine and isoleucine against H_2_O_2_-induced oxidative damage in MAC-T cells was investigated. Briefly, MAC-T cells exposed or free to H_2_O_2_ were incubated with different combinations of leucine and isoleucine. The cellular relative proliferation rate and viability, oxidative stress indicators, and inflammatory factors were determined by specific commercial kits. The genes related to barrier functions were measured by real-time quantitative PCR. The protein expression differences were explored by 4D label-free quantitative proteomic analyses and validated by parallel reaction monitoring. This finding will enhance our understanding of the protective mechanisms of leucine and isoleucine against oxidative damage.

## 2. Materials and Methods

### 2.1. Cell Culture

The MAC-T cells used in this study were donated by Professors Liu Jianxin and Liu Hongyun of Zhejiang University (Hangzhou, China). The cells were cultured for 15 passages and grown in Dulbecco's modified eagle medium F12 (DMEM/F12) (Gibco, Carlsbad, CA, USA) and supplemented with 10% fetal bovine serum (FBS) (Hyclone, Logan, UT, USA) and a 1% final concentration of antibiotics (penicillin and streptomycin) under 5% CO_2_ humidity at 37°C. Culturing was performed in a 25 cm^2^ culture flask (Corning Inc., NY, USA) containing 5 mL medium and a seeding number of 5 × 10^5^ cells/flask. The medium was changed every other day.

### 2.2. Oxidative Stress Model: Time of H_2_O_2_ Treatment

At a confluence of 70–80%, the cells were exposed to completed medium containing 0, 200, 400, 600, 800, 1000, or 1200 *μ*mol/L H_2_O_2_ (working concentration) for 2, 4, 6, 8, or 10 h, after which the cellular viability of the MAC-T cells was measured.

### 2.3. Oxidative Stress Model: Concentration of H_2_O_2_ Treatment

At a confluence of 70–80%, the cells were exposed to completed medium containing 0, 200, 400, 600, 800, 1000, or 1200 *μ*mol/L H_2_O_2_ (working concentration) for 6 h (based on the results of 2.2.), after which oxidative stress indicators of the MAC-T cells were measured.

### 2.4. Time and Concentration of Leucine Treatment

At a confluence of 70–80%, the cells were treated with 0.450, 0.675, 0.900, 1.125, or 1.350 mmol/L leucine (Sigma, St. Louis, USA) diluted in DMEM/F12 with antibiotics for 6, 12, 24, 48, or 72 h. The experimental treatments correspond to 1, 1.5, 2, 2.5, and 3 times the leucine concentration in DMEM/F12, which was 0.450 mmol/L. The relative proliferation rate of the MAC-T cells was measured.

### 2.5. Time and Concentration of Isoleucine Treatment

At a confluence of 70–80%, the cells were treated with 0.420, 0.630, 0.840, 1.050, or 1.260 mmol/L isoleucine (Sigma, St. Louis, USA) diluted in DMEM/F12 with antibiotics for 6, 12, 24, 48, or 72 h. The experimental treatments correspond to 1, 1.5, 2, 2.5, and 3 times the isoleucine concentration in DMEM/F12, which was 0.420 mmol/L. The relative proliferation rate of the MAC-T cells was measured.

### 2.6. Time and Concentration of the Combinations of Leucine and Isoleucine Treatment

Based on the leucine (2.4.) and isoleucine (2.5.) results, 24 h was selected as the treatment time and 9 concentrations of leucine and isoleucine combined were designed as follows: (1) 0.450 mmol/L leucine and 0.420 mmol/L isoleucine; (2) 0.450 mmol/L leucine and 0.630 mmol/L isoleucine; (3) 0.450 mmol/L leucine and 0.840 mmol/L isoleucine; (4) 0.675 mmol/L leucine and 0.420 mmol/L isoleucine; (5) 0.675 mmol/L leucine and 0.630 mmol/L isoleucine; (6) 0.675 mmol/L leucine and 0.840 mmol/L isoleucine; (7) 0.900 mmol/L leucine and 0.420 mmol/L isoleucine; (8) 0.900 mmol/L leucine and 0.630 mmol/L isoleucine; and (9) 0.900 mmol/L leucine and 0.840 mmol/L isoleucine. At a confluence of 70–80%, the cells were placed in DMEM/F12 with antibiotics containing different combinations of leucine and isoleucine for 24 h to determine the relative proliferation rate.

### 2.7. MAC-T Cells Treatment

Based on the above screening results, MAC-T cells were firstly cultured in completed medium with or without 600 *μ*mol/L H_2_O_2_ for 6 h and then incubated in DMEM/F12 medium (1% antibiotics) with different combinations of leucine and isoleucine for 24 h (Supplementary Table1).

### 2.8. Cellular Relative Proliferation Rate and Viability

A 200 *μ*L MAC-T cells suspension (5 × 10^4^ cells/mL) was seeded into the wells of a 96-well plate. After the cell confluence reached 70–80%, the completed medium was removed, and the cells were washed twice with cold PBS. Then, the cells were incubated in treated medium. The cellular viability was measured using a CCK-8 commercial kit (Cat No. BS350A; Biosharp, Guangzhou, China). The absorbance (OD value) was determined using an enzyme-labeling instrument (BioTek, Hong Kong, China) at a wavelength of 450 nm. The relative proliferation rate and cellular viability were calculated using the following formulas:

(1) $$\begin{array}{c} \text{Relative proliferation rate}=\frac{\text{OD test}}{\text{OD control}},\\ \text{Cellular viability}=\frac{\left(\text{OD test}-\text{OD blank}\right)}{\left(\text{OD control}-\text{OD blank}\right)\;}\times 100\%. \end{array}$$

### 2.9. Oxidative Stress Indicators and Inflammatory Factors

A 2 mL MAC-T cells suspension (5 × 10^4^ cells/mL) was seeded into the wells of a 6-well plate. After the cell confluence reached 70–80%, the completed medium was removed, and the cells were washed twice with cold PBS. Then, the cells were incubated with treated medium. The MDA content of the MAC-T cells was determined using a microplate method kit (Cat No. A003-4-1; Nanjing Jiancheng Biotechnology Institute, Nanjing, China). The total oxidant status (TOS, Cat No. BPE92369), total antioxidant status (TAS, Cat No. BPE92207), TNF-*α* (Cat No. BPE92091), IL-1*β* (Cat No. BPE92157), IL-6 (Cat No. BPE92153), and IL-10 (Cat No. BPE92159) of the MAC-T cells were detected using ELISA kits (Shanghai Lengton Bioscience Co., Ltd., Shanghai, China) following the manufacturer's instructions. Briefly, MAC-T cells were digested by trypsin and placed in centrifuge tubes. Then, the tubes were centrifuged at 2,000 rpm for 20 min, and the supernatant was collected. After the cells were counted, the cell suspension was adjusted with PBS to reach a cell concentration of 1 × 10^6^ cells/mL. Absorbance was detected at 530 (MDA) and 450 nm (TOS, TAS, TNF-*α*, IL-1*β*, IL-6, and IL-10) using an enzyme-labeling instrument (BioTek, Hong Kong, China).

### 2.10. Total RNA Extraction and Real-Time Quantitative PCR (RT-qPCR)

TRIzol reagent (Cat No. DP430; Tiangen Biotechnology Co., Ltd., Beijing, China) was used to extract the total RNA from the MAC-T cells following the manufacturer's instructions. Then, the quantity and quality of the total RNA were assessed using an ultraviolet-visible fluorescence spectrometer (Eppendorf, Hamburg, Germany). The samples with an A260/A280 ratio of 2.0 : 2.2 were used for subsequent PCR reactions. Finally, the total RNA was reverse transcribed into cDNA using a PrimeScript RT Master Mix kit (Cat No. KR116-01; Tiangen Biotechnology Co., Ltd., Beijing, China). The cDNA samples were stored at −20°C for further analysis.

The mRNA expressing certain genes, including claudin-1, occludin, and zonula occludens-1 (ZO-1), was evaluated by RT-qPCR. The primers of these genes and the housekeeping gene (*β*-actin) were synthesized by Sangon Biotechnology Co., Ltd. (Shanghai, China) (Table 1). RT-qPCR assays were conducted using a StepOnePlus Real-Time PCR System (Applied Biosystems, Carlsbad, CA, USA) using Talent qPCR PreMix kits (Cat No. FP209-01; Tiangen Biotechnology Co., Ltd., Beijing, China). The total reaction volume was 20 *μ*L: 10 *μ*L Talent qPCR premix (2 ×), 2 *μ*L ROX Reference Dye (50×), 4.8 *μ*L RNase-free ddH_2_O, 0.6 *μ*L of reverse primer (100 *μ*mol), 0.6 *μ*L of forward primer (100 *μ*mol), and 2 *μ*L cDNA. The reaction conditions were as follows: 95°C for 3 min; 40 cycles at 95°C for 5 s; and 60°C for 15 s. The cycle threshold values were normalized to *β*-actin. The relative mRNA expression levels of the selected genes were calculated using the 2^−*ΔΔ*Ct^ method [18].

### 2.11. 4D Label-Free Quantitative Proteomic Profiling

Four volumes of lysis buffer containing 8 mol/L urea and 1% protease inhibitor cocktail were added to MAC-T cells samples from each treatment group and then sonicated three times on ice using a high intensity ultrasonic processor (Scientz-5 T; Scientz, Ningbo, China). The supernatant was collected after centrifugation at 12,000 g for 10 min at 4°C. Then, the protein concentration was determined using a BCA kit (Cat No. A045-4-2; Beyotime Biotechnology, Shanghai, China) following the manufacturer's instructions.

The protein concentration of each sample was enzymatically hydrolyzed in equal quantities, and the volume was adjusted to be consistent with the lysate. Then, 20% trichloroacetic acid (Sigma, St. Louis, USA) was gradually added. The solution was mixed, vortexed, and incubated at 4°C for 2 h and then centrifuged at 4,500 g for 5 min. The supernatant was discarded, and the precipitate was washed with prechilled acetone three times. The acetone-precipitated protein pellets were suspended in 200 mmol/L TEAB (Sigma, St. Louis, USA). Then, trypsin (trypsin : proteins =1 : 50) (Promega, WI, USA) was added for digestion overnight. The solution was reduced with 5 mmol/L dithiothreitol (Sigma, St. Louis, USA) for 30 min at 56°C. Finally, iodoacetamide (Sigma, St. Louis, USA) was added to a final concentration of 11 mmol/L followed by incubation at room temperature in the dark for 15 min.

Liquid chromatography tandem mass spectrometry (LC-MS/MS) analysis was performed. Digested peptides were dissolved by LC mobile phase A containing 0.1% formic acid and 2% acetonitrile and then separated by a NanoElute ultraperformance LC system (Waters Corporation, MA, USA). The elution gradient was as follows: 6–24% solvent B containing 0.1% formic acid in 100% acetonitrile for 70 min, 24–32% for 14 min, 32–80% for 3 min, and finally kept at 80% for 3 min. All operations were performed on a NanoElute ultraperformance LC system at a constant flow rate of 450 nL/min. Peptides were separated by an ultrahigh performance liquid phase system subjected to a capillary ion source and then analyzed by timsTOF Pro MSP (Bruker Daltonics, Bremen, Germany); the electrospray voltage was 1.75 kV. The peptide parent ions and their secondary fragments were detected and analyzed using high-resolution TOF. The secondary MS scanning range was 100–1700 m/z. Data acquisition on the timsTOF Pro was collected using the parallel accumulation serial fragmentation (PASEF) acquisition mode. After the first MS stage, the second MS stage (charge number of the parent ions was 0–5) was recorded using the 10 PASEF mode. A dynamic exclusion time of 30 s was used for the MS/MS scan. When the threshold of the protein relative expression ratio increased by ≥1.5-fold or decreased by ≤0.6-fold, the result was considered significant.

A bioinformatics analysis using 4 comparison groups (H1L1I vs. control, H1L2I vs. H1L1I, H2L1I vs. H1L1I, and H2L1.5I vs. H1L1I) was performed. Functional annotations of the quantified proteins were acquired using the Gene Ontology Annotation (GOA) Database (EMBL-EBI, Hinxton, Cambridgeshire, UK) and Kyoto Encyclopedia of Genes and Genomes (KEGG) pathway analysis databases [19]. For the GO annotations and protein pathways, a two-tailed Fisher's exact test was used to test the enrichment of differentially expressed (DE) proteins against all identified proteins using a corrected *p* value < 0.05, which was considered significant. The ggplot2 package in R software was used to construct a volcano map of the DE proteins with a 1.5-times fold change. Hierarchical clustering based on different protein pathways was conducted using the collated categories obtained after enrichment and after *p* values were filtered, which were enriched in at least 1 cluster with *p* < 0.05. Then, the filtered *p* value matrix was transformed using the function:

(2) $$\begin{array}{c} x=-\log10\left(p-\text{value}\right). \end{array}$$

The *x* values were *z*-transformed for each functional category. Then, the *z*-scores were clustered by one-way hierarchical clustering (Euclidean distance, average linkage clustering) using Genesis [20].

### 2.12. Parallel Reaction Monitoring (PRM) Validation

The expression levels of the candidate proteins screened by 4D label-free quantitative proteomic analyses were verified by quantification by parallel reaction monitoring (PRM) technique [21]. The specific PRM validation procedure was obtained from Jingjie PTM BioLab Co. Ltd. (Hangzhou, China) as follows: the protein solution was reduced with 5 mmol dithiothreitol for 30 min at 56°C and alkylated with 11 mmol/L iodoacetamide for 15 min at room temperature in darkness.. The protein sample was then diluted to urea concentration less than 2 mol/L. Finally, trypsin was added at 1 : 50 trypsin-to-protein mass ratio for the first digestion overnight and 1 : 100 trypsin-to-protein mass ratio for a second 4 h digestion. Then, the analysis was performed using LC-MS/MS. The tryptic peptides were dissolved in 0.1% formic acid (solvent A), directly loaded onto a homemade reversed-phase analytical column. The gradient was comprised of an increase from 6% to 23% solvent B (0.1% formic acid in 98% acetonitrile) over 38 min, 23% to 35% in 14 min climbing to 80% in 4 min and then holding at 80% for the last 4 min, all at a constant flow rate of 700 nL/min on an EASYnLC 1000 UPLC system. The peptides were subjected to NSI source followed by MS/MS in Q Exactive™ Plus (Thermo) coupled online to the UPLC. The electrospray voltage applied was 2.0 kV. The m/z scan range was 350 to 1000 for full scan, and intact peptides were detected in the Orbitrap at a resolution of 35,000. Peptides were then selected for MS/MS using NCE setting as 27, and the fragments were detected in the Orbitrap at a resolution of 17,500. A data-independent procedure that alternated between one MS scan followed by 20 MS/MS scans. Automatic gain control (AGC) was set at 3E6 for full MS and 1E5 for MS/MS. The maximum IT was set at 20 ms for full MS and auto for MS/MS. The isolation window for MS/MS was set at 2.0 m/z. The resulting MS data were processed using Skyline (v.3.6). In the peptide settings, the enzyme was set as Trypsin [KR/P] and max missed cleavage was set as 2. The peptide length was set as 8-25. Variable modification was set as Carbamidomethyl on Cys and oxidation on Met, and max variable modifications was set as 3. In the transition settings, precursor charges were set as 2, 3, ion charges were set as 1, 2, and ion types were set as b, y, p. The productions were set as from ion 3 to last ion, and the ion match tolerance was set as 0.02 Da.

### 2.13. Statistical Analysis

The data are presented as the mean ± standard deviation (SD). Statistical analyses were conducted using SPSS version 22.0 (IBM Inc., Beijing, China). The data between different groups were compared by a one-way analysis of variance (ANOVA) followed by Duncan's post hoc multiple comparisons test. For the different statistical tests, a significance threshold of *p* < 0.05 was used.

## 3. Results

### 3.1. Oxidative Stress Model: Effects of the Different H_2_O_2_ Concentrations for Different Times on the Cellular Viability of MAC-T Cells

**Figure 1:**
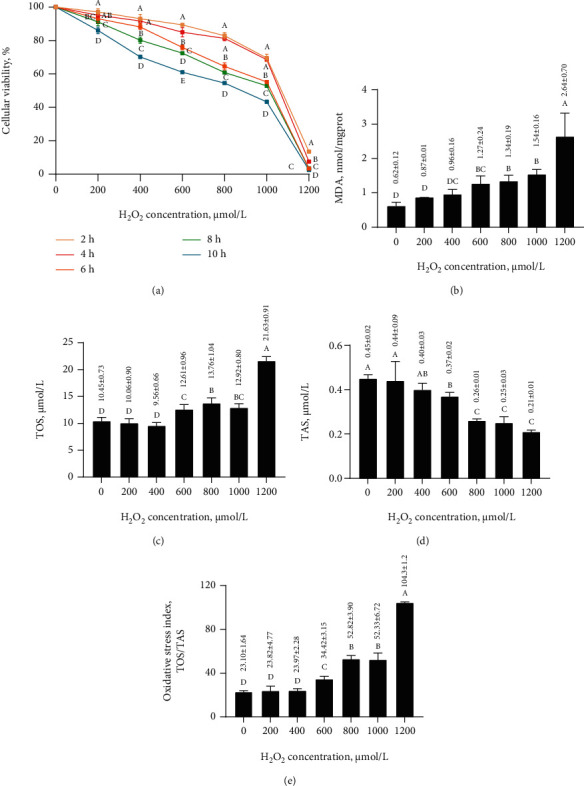
Oxidative stress model. (a) Effects of H_2_O_2_ on the cellular viability of MAC-T cells. Different letters (A–E) indicate significant differences at the same concentration (*p* < 0.05). Effects of different H_2_O_2_ concentrations for 6 h on oxidative stress indicators in MAC-T cells: MDA (b), TOS (c), TAS (d), and TOS/TAS (e). Different letters (A–D) among treatments indicate significant differences (*p* < 0.05).

The cellular viability of cells gradually decreased as the H_2_O_2_ concentration increased over time (Figure 1(a) and Supplementary Table 2). Cellular viability in 600 and 1200 *μ*mol/L H_2_O_2_ decreased significantly (*p* < 0.05) after 4 h (84.99 ± 2.78% and 7.58 ± 0.18%, respectively) but decreased significantly (*p* < 0.05) in 200, 400, 800, and 1000 *μ*mol/L H_2_O_2_ after 6 h (93.08 ± 2.26%, 88.16 ± 1.57%, 64.58 ± 2.11%, and 55.20 ± 0.73%, respectively). Thus, the H_2_O_2_ treatment time of 6 h was selected for the oxidative stress model.

### 3.2. Oxidative Stress Model: Effects of Different H_2_O_2_ Concentrations for 6 h on the Oxidative Stress Indicators in MAC-T Cells

H_2_O_2_ significantly increased (*p* < 0.05) the TOS/TAS, MDA, and TOS but significantly decreased (*p* < 0.05) the TAS in MAC-T cells treated with ≥600 *μ*mol/L H_2_O_2_ when compared to 0 *μ*mol/L H_2_O_2_ (TOS/TAS, 34.42 ± 3.15 vs. 23.10 ± 1.64; MDA, 1.27 ± 0.24 vs. 0.62 ± 0.12 nmol/mgprot; TOS, 12.61 ± 0.96 vs. 10.45 ± 0.73 *μ*mol/L; and TAS, 0.37 ± 0.02 vs. 0.45 ± 0.02 *μ*mol/L) (Figures 1(b)–1(e)). Thus, 600 *μ*mol/L H_2_O_2_ was used for the oxidative stress model.

### 3.3. Effects of Different Leucine Concentrations for Different Times on the Relative Proliferation Rate of MAC-T Cells

**Figure 2:**
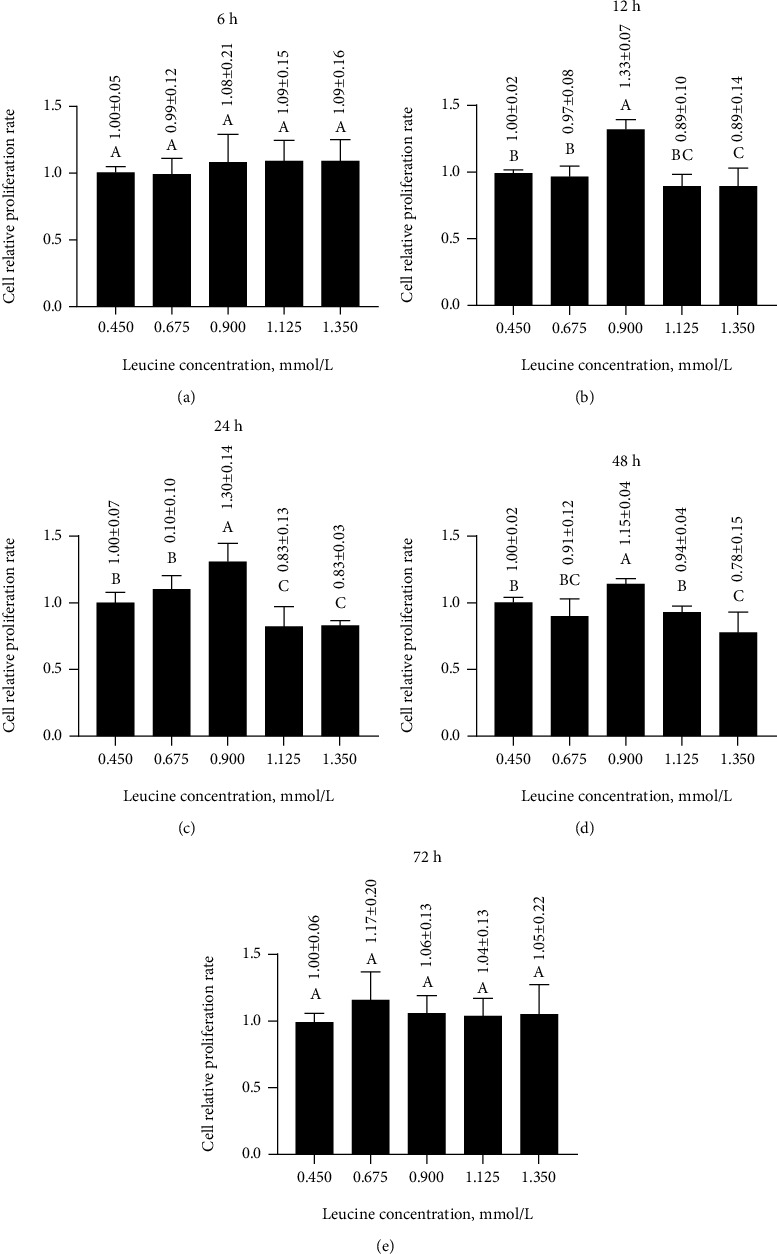
Effects of different leucine concentrations for 6 (a), 12 (b), 24 (c), 48 (d), or 72 h (e) on the relative proliferation rate of MAC-T cells. Different letters (A–C) among treatments indicate significant differences (*p* < 0.05).

After 6 or 72 h, no significant differences were detected for the relative proliferation rate among leucine concentration treatments (Figures 2(a) and 2(e)). After 12 h, the relative proliferation rate significantly increased (*p* < 0.05) and peaked at 1.33 ± 0.07 under 0.900 mmol/L leucine (Figure 2(b)) and then significantly decreased (*p* < 0.05) to 0.84 ± 0.14 under 1.350 mmol/L leucine. Similarly, after 24 h, the relative proliferation rate significantly increased (*p* < 0.05) to 1.30 ± 0.14 under 0.900 mmol/L leucine (Figure 2(c)), followed by a significant decrease (*p* < 0.05) to 0.83 ± 0.03 under 1.350 mmol/L leucine. Likewise, after 48 h, despite an initial significant increase (*p* < 0.05), the relative proliferation rate significantly decreased (*p* < 0.05) from its peak under 0.900 mmol/L leucine at 1.15 ± 0.04 to 0.78 ± 0.15 under 1.350 mmol/L leucine (Figure 2(d)). Thus, the leucine concentrations of 0.450, 0.675, and 0.900 mmol/L and the treatment times of 12, 24, 48 h were selected for further analysis.

### 3.4. Effects of Different Isoleucine Concentrations for Different Times on the Relative Proliferation Rate of MAC-T Cells

**Figure 3:**
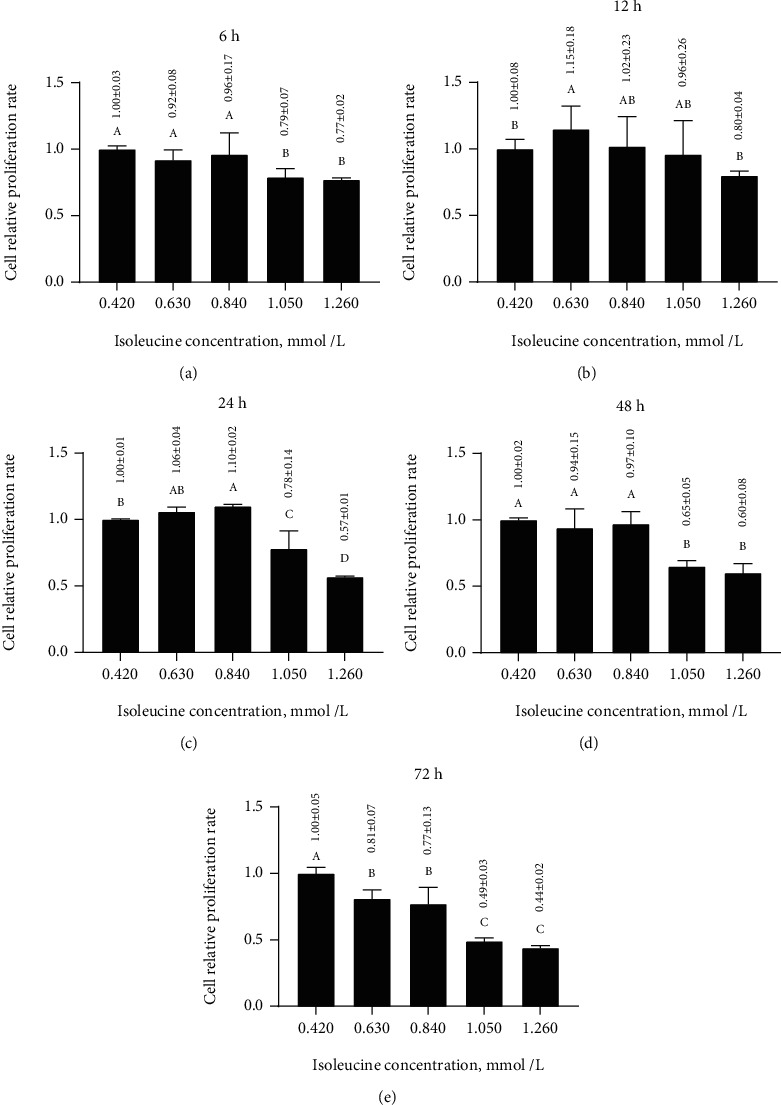
Effects of different isoleucine concentrations for 6 (a), 12 (b), 24 (c), 48 (d), or 72 h (e) on the relative proliferation rate of MAC-T cells. Different letters (A–D) among treatments indicate significant differences (*p* < 0.05).

Among the isoleucine concentration treatments, the relative proliferation rate after 6 and 48 h exhibited similar trends (Figure 3(a) and 3(d)). After stabilizing from 0.420 to 0.840 mmol/L isoleucine, the relative proliferation rate significantly decreased (*p* < 0.05) to 0.77 ± 0.02 after 6 h and 0.60 ± 0.08 after 48 h under 1.260 mmol/L isoleucine. However, after 12 h (Figure 3(b)), the relative proliferation rate peaked at 1.15 ± 0.18 under 0.630 mmol/L isoleucine before decreasing significantly (*p* < 0.05) to 0.80 ± 0.04 under 1.260 mmol/L isoleucine. Similarly, after 24 h (Figure 3(c)), the relative proliferation rate significantly increased (*p* < 0.05) from 1.00 under 0.420 mmol/L isoleucine and peaked at 1.10 ± 0.02 under 0.840 mmol/L isoleucine before significantly decreasing (*p* < 0.05) to 0.57 ± 0.01 under 1.260 mmol/L isoleucine. After 72 h, the relative proliferation rate decreased significantly (*p* < 0.05) from 1.00 under 0.420 mmol/L isoleucine to 0.44 ± 0.02 under 1.260 mmol/L isoleucine (Figure 3(e)). Thus, the isoleucine concentrations of 0.420, 0.630, and 0.840 mmol/L and the treatment time of 24 h were selected for further analysis.

### 3.5. Effects of Different Concentrations of Leucine and Isoleucine Combined after 24 h on the Relative Proliferation Rate of MAC-T Cells

**Figure 4:**
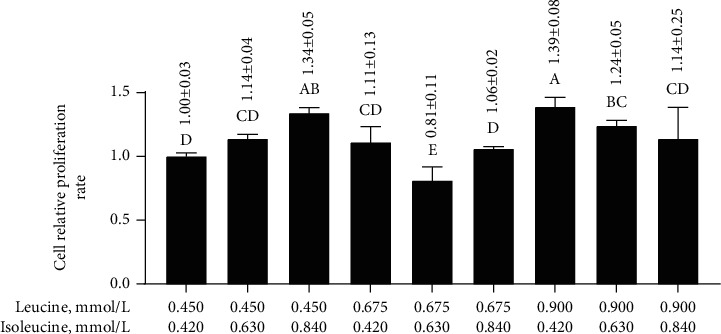
Effects of different combinations of leucine and isoleucine for 24 h on the relative proliferation rate of MAC-T cells. Different letters (A–E) among treatments indicate significant differences (*p* < 0.05).

The relative proliferation rates under 0.450 mmol/L leucine and 0.840 mmol/L isoleucine, 0.900 mmol/L leucine and 0.420 mmol/L isoleucine, and 0.900 mmol/L leucine and 0.630 mmol/L isoleucine were significantly higher (*p* < 0.05) than 0.450 mmol/L leucine and 0.420 mmol/L isoleucine (Figure 4). Thus, these combined concentrations of leucine and isoleucine were selected to further investigate the protective mechanisms of leucine and isoleucine against H_2_O_2_-induced oxidative damage in MAC-T cells.

### 3.6. Effects of Leucine and Isoleucine on the Relative Proliferation Rate and Oxidative Stress Indicators in MAC-T Cells due to H_2_O_2_-Induced Oxidative Damage

**Figure 5:**
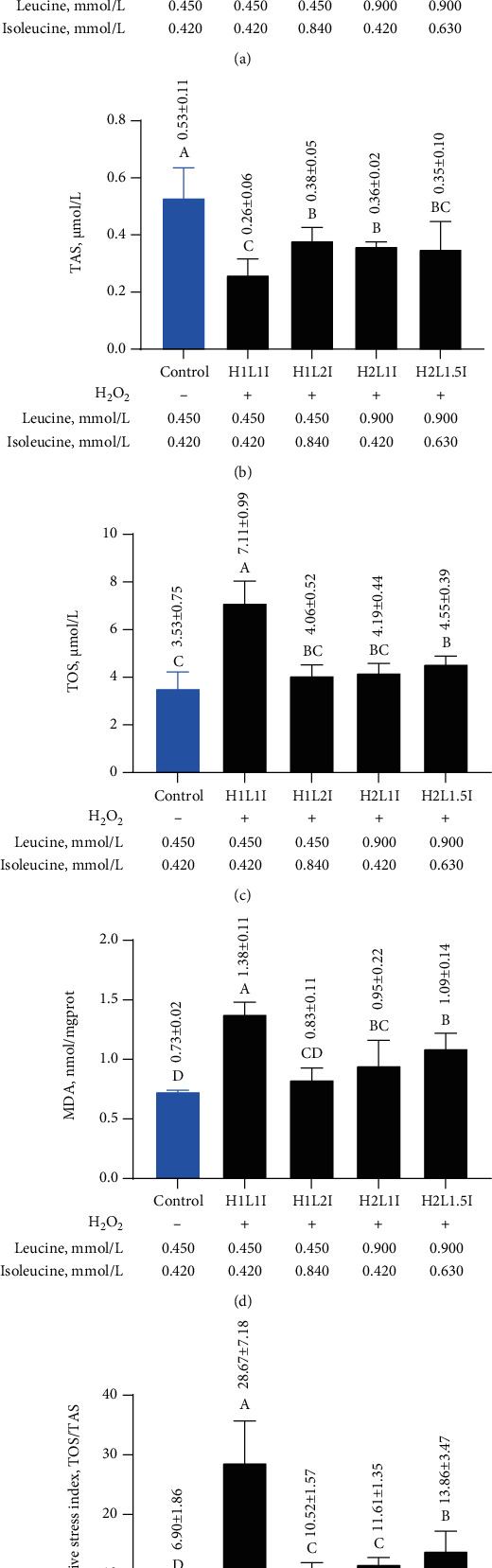
Effects of different combinations of leucine and isoleucine for 24 h on the relative proliferation rate (a) and oxidative stress indicators (TAS (b), TOS (c), MDA (d), and TOS/TAS (e)) in MAC-T cells due to H_2_O_2_-induced oxidative damage. Different letters (A–D) among treatments indicate significant differences (*p* < 0.05).

The relative proliferation rate and TAS significantly decreased (*p* < 0.05), while TOS, MDA, and TOS/TAS significantly increased (*p* < 0.05) in the H1L1I group when compared to the control group, indicating that H_2_O_2_-induced oxidative damage had occurred in MAC-T cells (Figure 5). Compared to the H1L1I group, the relative proliferation rate significantly increased (*p* < 0.05) in the H1L2I, H2L1I, and H2L1.5I groups by 23.08%, 34.62%, and 24.36%, respectively (Figure 5(a)). However, TAS significantly increased (*p* < 0.05) in the H1L2I and H2L1I groups by 46.15% and 38.46%, respectively (Figure 5(b)), but not in the H2L1.5I group. TOS and MDA in the H1L2I, H2L1I, and H2L1.5I groups were significantly lower (*p* < 0.05) than the H1L1I group (Figures 5(c) and 5(d)). Additionally, the MDA content in the H1L2I group was significantly lower (*p* < 0.05) than in the H2L1.5I group. Compared to the H1L1I group, TOS/TAS significantly decreased (*p* < 0.05) in the H1L2I, H2L1I, and H2L1.5I groups (Figure 5(e)). However, TOS/TAS significantly increased (*p* < 0.05) by 25.20% in the H2L1.5I group when compared to the H1L2I and H2L1I groups.

### 3.7. Effects of Leucine and Isoleucine on Inflammatory Factors in MAC-T Cells due to H_2_O_2_-Induced Oxidative Damage

**Figure 6:**
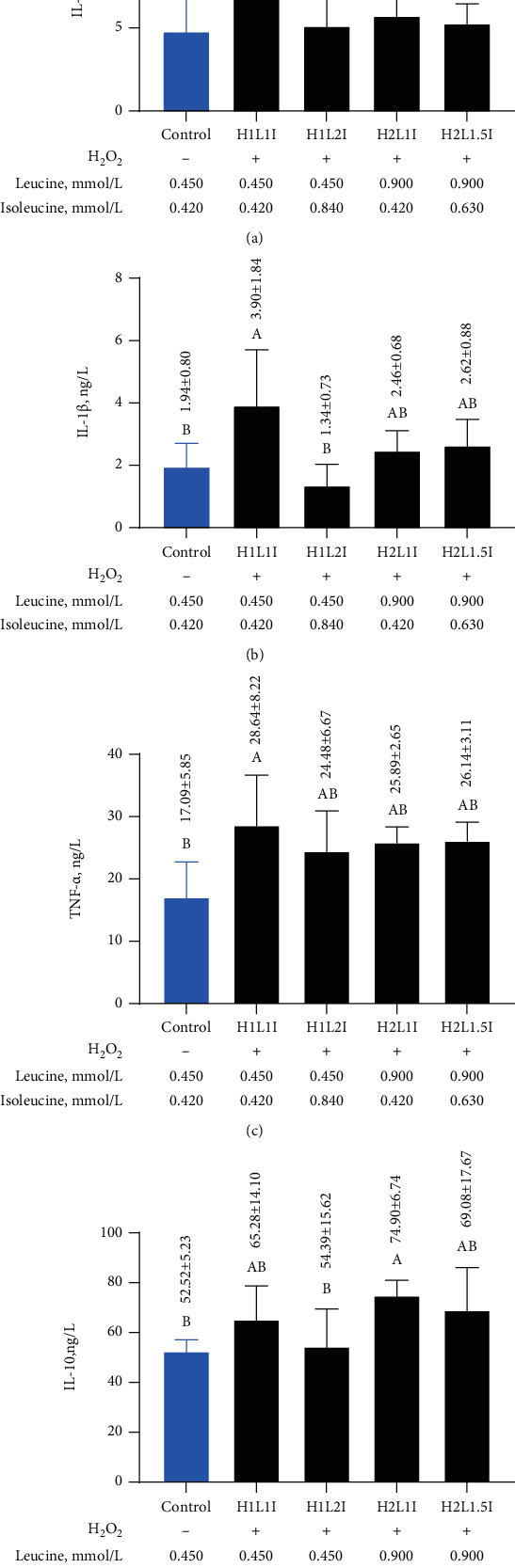
Effects of different combinations of leucine and isoleucine for 24 h on IL-6 (a), IL-1*β* (b), TNF-*α* (c), and IL-10 (d) in MAC-T cells due to H_2_O_2_-induced oxidative damage. Different letters (A, B) among treatments indicate significant differences (*p* < 0.05).

The TNF-*α*, IL-1*β*, and IL-6 levels in MAC-T cells significantly increased (*p* < 0.05) in the H1L1I group when compared to the control group (Figure 6). However, this upward trend was suppressed to a certain extent by increased concentrations of leucine or isoleucine. Compared to the H1L1I group, IL-6 significantly decreased (*p* < 0.05) by 48.24% in the H1L2I, H2L1I, and H2L1.5I groups, while 1 L-1*β* significantly decreased (*p* < 0.05) in the H1L2I group by 65.64% (Figures 6(a) and 6 (b)). However, leucine and/or isoleucine intervention did not induce a significant change in the TNF-*α* level (Figure 6(c)). Additionally, there were no significant differences detected in IL-10 between the H1L1I and control groups, but the IL-10 level in the H2L1I group was significantly higher (*p* < 0.05) than that in the H2LI and control groups (Figure 6(d)).

### 3.8. Effects of Leucine and Isoleucine on Barrier Functions in MAC-T Cells due to H_2_O_2_-Induced Oxidative Damage

**Figure 7:**
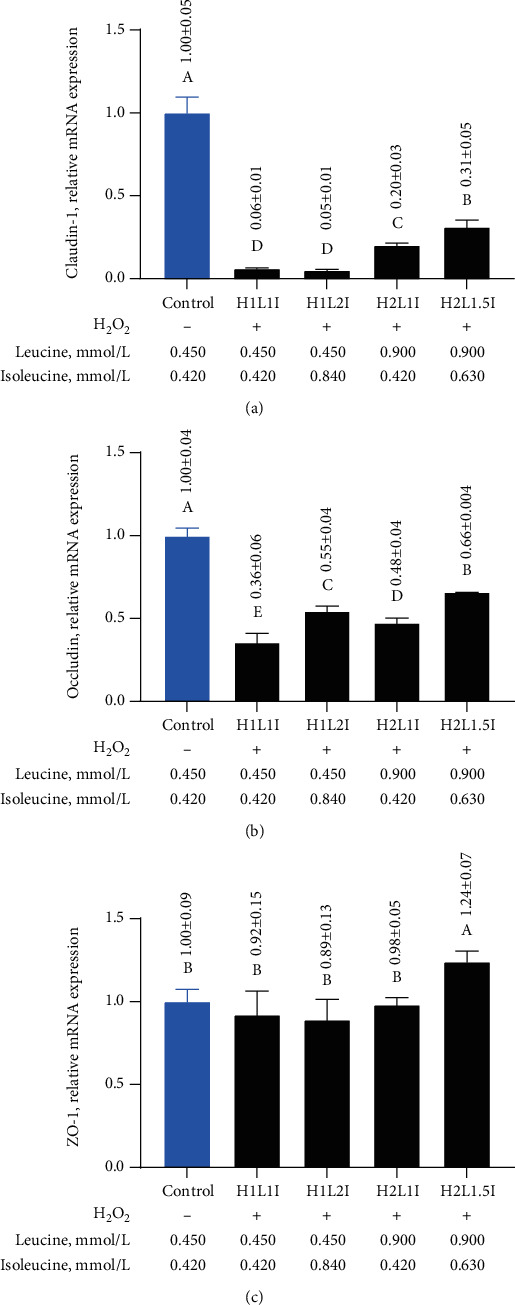
Effects of different combinations of leucine and isoleucine for 24 h on the mRNA expression levels of claudin-1 (a), occludin (b), and ZO-1 (c) in MAC-T cells due to H_2_O_2_-induced oxidative damage. Different letters (A–E) among treatments indicate significant differences (*p* < 0.05).

To assess the effects of leucine and isoleucine on the barrier functions in MAC-T cells due to H_2_O_2_-induced oxidative damage, the mRNA expression levels of claudin-1, occludin, and ZO-1 were determined. Specifically, the mRNA expression levels of claudin-1 and occludin significantly decreased (*p* < 0.05) in MAC-T cells due to oxidative stress (Figures 7(a) and 7 (b)). When compared to the H1L1I group, leucine significantly increased (*p* < 0.05) the mRNA expression levels of claudin-1 and occludin and isoleucine significantly increased (*p* < 0.05) the mRNA expression level of occludin (*p* < 0.05). Additionally, combined leucine and isoleucine significantly increased (*p* < 0.05) the mRNA expression levels of claudin-1, occludin, and ZO-1 (Figure 7(c)).

### 3.9. Protein Expression Differences

**Figure 8:**
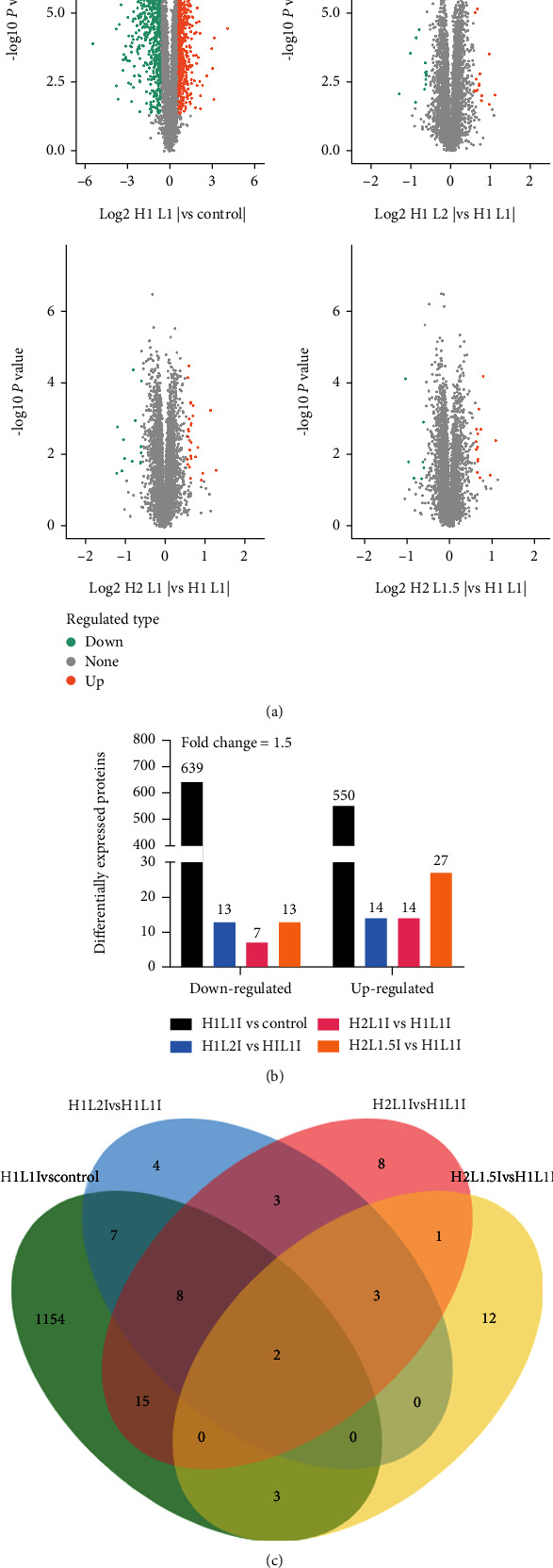
Distribution of proteins identified in MAC-T cells of the control, H1L1I, H1L2I, H2L1I, and H2L1.5I groups. (a) Volcano plot of differentially expressed (DE) proteins. The horizontal coordinate indicates the fold change (log2 transformation) and the vertical coordinate indicates the *p*-values (log10 transformation). Red dots indicate upregulation and green dots indicate downregulation. (b) Upregulated and downregulated DE proteins. (c) Venn diagram of DE proteins identified in MAC-T cells due to H_2_O_2_-induced oxidative damage (fold change ≥ 1.5).

Proteomic data of the MAC-T cells in the five groups are shown in Figure 8. A total of 1220 quantifiable DE proteins were successfully identified in this study. Based on the volcano plot (red, upregulated; green, downregulated), the number of downregulated or upregulated proteins in the H1L2I, H2L1I, and H2L1.5I groups overlapped and each group had unique proteins (Figure 8(a)). Interestingly, the amount of downregulated proteins in the H2L1I group was two times lower than in the H1L2I and H2L1.5I groups, while the amount of upregulated DE proteins in the H2L1.5I group was two times higher than in the H1L2I and H2L1I groups (Figure 8(b)). There were 1189 DE proteins identified in the H1L1I group when compared to the control group. Compared to the H1L1I group, the same 5 proteins were identified in the H1L2I, H2L1I, and H2L1.5I groups, while 11, 23, and 15 unique proteins were identified in these same groups, respectively (Figure 8(c)).

### 3.10. GO Enrichment of Differentially Quantified Proteins

**Figure 9:**
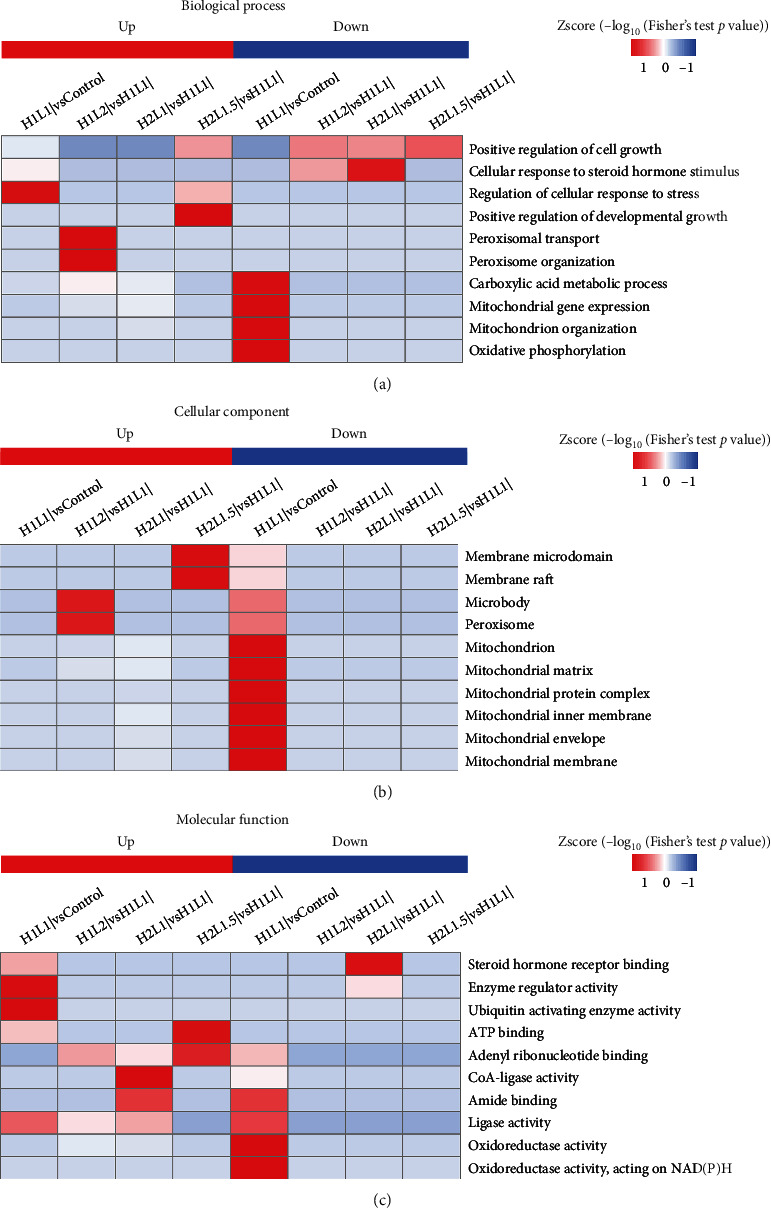
Cluster analysis heat map based on GO enrichment classifications: (a) biological process (BP); (b) cellular component (CC); and (c) molecular function (MF). The DE proteins among the comparison groups were subjected to GO enrichment, and a cluster analysis was performed to determine the correlative relationships among the DE protein functions. The horizontal direction indicates the enrichment test results of different groups, and the vertical direction indicates the description of DE enrichment-related functions. Different sets of DE proteins and color blocks correspond with the functional descriptions and indicate the degree of enrichment, where red indicates stronger enrichment (the deeper the red, the stronger the enrichment) and blue indicates weaker enrichment (the lighter the blue, the weaker the enrichment).

Following GO classification with biological process (BP), cellular component (CC), and molecular function (MF), a cluster analysis was conducted to compare the functional correlations between DE proteins in the experimental groups; the results are displayed in a heat map (Figure 9). When cells were subjected to oxidative stress, some downregulated proteins with functional annotations in the H1L1I group were identified, including carboxylic acid metabolic process and mitochondrial gene expression in BP (Figure 9(a)), mitochondrion and mitochondrial matrix in CC (Figure 9(b)), and oxidoreductase activity, oxidoreductase activity, and activity on NAD (P) H in MF (Figure 9(c)). Several upregulated proteins with functional annotations were identified in the H1L1I group, including regulation of cellular response to stress in BP and enzyme regulator activity and ubiquitin activating enzyme activity in MF. Furthermore, when cells subjected to H_2_O_2_-induced oxidative damage were treated with leucine or isoleucine, unique proteins were expressed in each group. The H1L2I group contained several unique proteins with functional annotations including peroxisomal transport and peroxisome organization in BP and microbody and peroxisome in CC. The H2L1I group contained unique proteins with functional annotations including steroid hormone receptor binding, enzyme regulator activity, CoA-ligase activity, and amide binding in MF. The H2L1.5I group had unique proteins with functional annotations including positive regulation of developmental growth and regulation of cellular response to stress in BP, membrane microdomain, membrane raft in CC, and ATP binding in MF. These results suggested that the intervention of leucine, isoleucine, or their combinations improved the condition of MAC-T cells subjected to oxidative stress, which was achieved through unique mechanisms.

### 3.11. Specific Regulation Pathways of Leucine and Isoleucine against H_2_O_2_-Induced Oxidative Damage in MAC-T Cells

**Figure 10:**
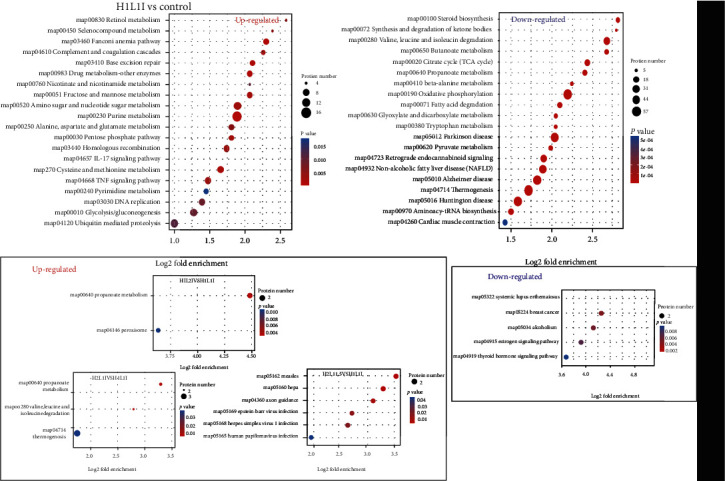
Bubble chart of DE proteins enriched in KEGG pathways. The bubble size represents the number of DE proteins in the enriched pathway terms, and the bubble color represents the *p* value.

To further explore the regulation pathways of leucine and isoleucine against H_2_O_2_-induced oxidative damage in MAC-T cells, a KEGG pathway enrichment analysis was conducted. Most of the pathways were significantly altered after MAC-T cells were subjected to oxidative stress (Figure 10). For example, in the H1L1I group, the map 04657 IL-17 and map 04668 TNF signaling pathways were significantly upregulated (*p* < 0.05), while the map 00280 valine, leucine, and isoleucine degradation, map 00640 propanoate metabolism, and map 04714 thermogenesis pathways were significantly downregulated (*p* < 0.05). When compared to the H1L1I group, the upregulation of a common pathway (map 00640 propanoate metabolism) occurred in the H1L2I and H2L1I groups, while unique pathways in the H1L2I (map 04146 peroxisome) and H2L1I groups (map 04714 thermogenesis and map 00280 valine, leucine, and isoleucine degradation) were upregulated; these pathways were not identified in the H2L1.5I group. Despite six upregulated pathways in the H2L1.5I group, these pathways were not detected when compared to the H1L1I and control groups. No significantly downregulated pathways were detected in the H1L2I or H2L1.5I groups. Although five downregulated pathways existed in the H2L1I group, these were not associated with H_2_O_2_-induced oxidative damage.

A total of 2, 2, 2, and 3 proteins were significantly upregulated (*p* < 0.05) in the propanoate metabolism; peroxisome; valine, leucine and isoleucine degradation; and thermogenesis pathways, respectively (Tables 2 and 3). Specifically, the expression of methylmalonate-semialdehyde dehydrogenase [acylating], mitochondrial (ALDH6A1) and propionate-CoA ligase (ACSS2) in propanoate metabolism, ALDH6A1, and isobutyryl-CoA dehydrogenase, mitochondrial (ACAD8) in valine, leucine, and isoleucine degradation, and protein arginine methyltransferase NDUFAF7 (NDUFAF7), NADH dehydrogenase [ubiquinone] 1 alpha subcomplex assembly factor 4 (NDUFAF4), and ATP synthase subunit f, mitochondrial (ATP5MF) in thermogenesis was significantly upregulated (*p* < 0.05) after leucine intervention (Table 2). After isoleucine intervention, the expression of ALDH6A1 and ACSS2 in propanoate metabolism and peroxisomal membrane protein PEX16 (PEX16) and phytanoyl-CoA dioxygenase, peroxisomal (PHYH) in peroxisome was significantly upregulated (*p* < 0.05) (Table 3).

### 3.12. PRM Protein Expression Quantities

**Figure 11:**
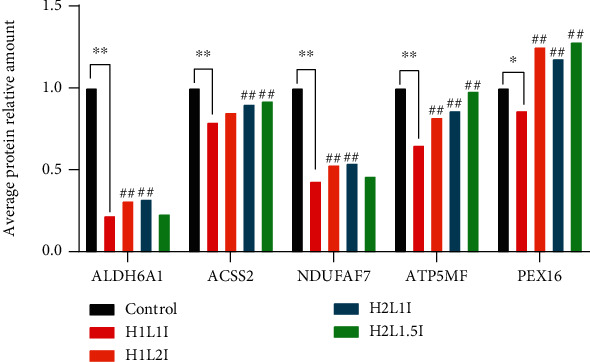
PRM protein expression quantities of the candidate proteins which were ALDH6A1, ACSS2, NDUFAF7, ATP5MF, and PEX16. ∗ indicates *p* < 0.05 and ∗∗ indicates *p* < 0.01 vs. control group. ## indicates *p* < 0.01 vs. H1L1I group.

The 8 target proteins shown in Tables 2 and 3 were selected for verification using PRM validation. Five of them named ALDH6A1, ACSS2, NDUFAF7, ATP5MF, and PEX16 were quantified as shown in Figure 11. After normalizing, the results of the relative quantitative expression showed that the 5 candidate proteins exhibited similar trends of those observed in the 4D label-free quantitative proteomic profiling results, which supports the plausibility and reliability of the proteomics data.

**Figure 12:**
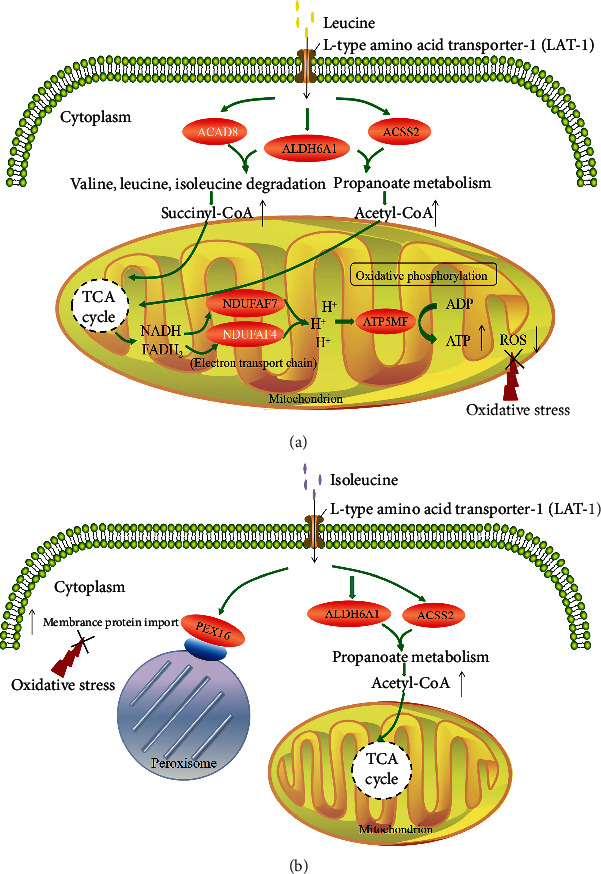
The protective mechanism of leucine (a) and isoleucine (b) against H_2_O_2_-induced oxidative damage in bovine mammary epithelial cells. Genes in black was validated in PRM and 4D label-free quantitative proteomic analysis, while genes in white was only validated in 4D label-free quantitative proteomic analysis.

Therefore, based on all above data, the protective mechanism of leucine and isoleucine against H_2_O_2_-induced oxidative damage in bovine mammary epithelial cells was outlined in Figure 12. In general, the intervention of leucine can generate more ATP for energy supplementation (Figure 12(a)); the intervention of isoleucine can improve the deficit in peroxisome transport and promote acetyl-CoA production, for MAC-T cells due to H_2_O_2_-induced oxidative damage (Figure 12(b)).

## 4. Discussion

Free radical production plays an essential role in normal metabolism, including hydrogen peroxide, superoxide radicals, and hydroxyl radicals [22]. Excessive free radical formation may lead to oxidative stress [23]. How to effectively alleviate oxidative stress damage to the body caused by free radicals has become an urgent problem. The balance between the oxidant and antioxidant defenses systems is important. Previously, leucine and isoleucine supplementation was found to increase antioxidation and reduce oxidative stress in the body [24–26]. H_2_O_2_ is the main product of oxidative stress [27]. Therefore, in this study, we induced oxidative stress *in vitro* by treating MAC-T cells with H_2_O_2_. Our findings suggested that leucine and isoleucine alleviated oxidative stress through certain pathways, which furthers our understanding of the antioxidant effects of certain nutrients.

Oxidative stress and apoptosis are considered effective immune defense mechanisms when the body is subjected to various harmful stimuli [28]. Sordillo and Aitken [2] found that a high MDA content indicated an imbalance between the level of oxidative stress and strength of the antioxidant defense system in dairy cows, which can increase the risk of disease. Jin et al. [29] found that the activation of endogenous ROS production in MAC-T cells subjected to H_2_O_2_ resulted in cumulative oxidative damage to cellular components, altered cellular functions, and apoptotic cell death. We also observed an increase in TOS and MDA, as well as a decrease in the relative proliferation rate and TAS when MAC-T cells were treated with H_2_O_2_, indicating that these cells were subjected to oxidative stress. Leucine and isoleucine acting as antioxidants have been reported [24–26]. Similarly, we found that they played antioxidative roles in MAC-T cells by increasing the relative proliferation rate and TAS, as well as decreasing TOS and MDA.

The KEGG pathway enrichment analysis results indicated that leucine intervention positively regulated the propanoate metabolism; valine, leucine, and isoleucine degradation; and thermogenesis pathways in MAC-T cells after oxidative damage. The upregulation of ACAD8 and ALDH6A1 expression in the valine, leucine, and isoleucine degradation pathway was observed, which promotes valine metabolism and generates succinyl-CoA [30, 31]. Succinyl-CoA enters the tricarboxylic acid (TCA) cycle to produce ATP [32]. Additionally, ALDH6A1 was found to be involved in the propanoate metabolism pathway to generate acetyl-CoA, which is an essential intermediate metabolite that enters the TCA cycle and is oxidized to yield energy [32]. The TCA cycle and electron transport chain are two main components that determine energy metabolism [33]. The expression levels of NDUFAF7, NDUFAF4, and ATP5MF in the electron transport chain (thermogenesis-related pathway) were upregulated, leading to electron coupling and oxidative phosphorylation to form ATP [34]. Consequently, generating more ATP for energy supplementation may be a potential mechanism by which leucine counters H_2_O_2_-induced oxidative damage in MAC-T cells. Additionally, isoleucine positively regulated the propanoate metabolism and peroxisome pathways in MAC-T cells after oxidative damage. Peroxisomes are small membrane-bound organelles that play diverse roles in the cellular metabolism, including the conversion of hydrogen peroxide to nontoxic forms [35, 36]. Mammalian Pex16 is an integral membrane protein that plays a role in the early stages of peroxisome biogenesis and promotes the formation of peroxisomes [36]. The upregulation of Pex16 expression was detected in the peroxisome pathway, which thereby facilitated the membrane protein transport of peroxisomes. Thus, improving the deficit in peroxisome transport and promoting acetyl-CoA production may be potential mechanisms by which isoleucine counters H_2_O_2_-induced oxidative damage in MAC-T cells. Interestingly, no DE proteins were enriched in the KEGG pathways associated with oxidative stress in MAC-T cells after oxidative damage when leucine and isoleucine concentrations were simultaneous increased. In addition, the H2L1.5I group had a higher TOS/TAS than the H1L2I and H2L1I groups. These results may be related to the concentrations and proportions of leucine and isoleucine or their interactions, as BCAAs share the same transporter on the cell membrane [37, 38]. However, further research is required to clarify and expand upon these findings.

Oxidative stress can activate the inflammation signaling pathway by activating pro-inflammatory cytokines, such as TNF-*α*, IL-1*β*, and IL-6 [39], thereby triggering excessive inflammatory and immune responses in dairy cows, which can result in certain diseases, including mastitis and abnormalities of the glucose and lipid metabolism [40, 41]. J. Lee et al. [42] reported that IL-6 mRNA expression decreased after leucine or isoleucine supplementation in the microglial cells of mice; the expression of proinflammatory cytokines also decreased as the concentrations of leucine, isoleucine, and valine increased, suggesting that an antagonistic effect among these BCAAs did not exist within the inflammatory response mechanism. Our study found similar results. IL-10 is an important anti-inflammatory cytokine that regulates the function of inflammatory immune cells [43]. In the present study, the intervention of leucine and/or isoleucine did not significantly affect IL-10 expression in MAC-T cells after oxidative damage, which corroborates the notion that different BCAAs do not alter the synthesis of IL-10 by macrophages [12].

Oxidative stress can affect barrier functions, leading to increased cell permeability [44]. Tight junction proteins are adhesive junction molecules that link epithelial cells together, including the occludin, ZO, and claudin protein families [45], which are involved in the regulation of cell permeability by promoting junction tightening [46]. When cells are subjected to oxidative stress, the mRNA expression levels of tight junction proteins significantly decrease [47], which is consistent with our results. Our data suggested that leucine or isoleucine treatment alleviated oxidative stress, which improved the mRNA expression levels of tight junction proteins, thereby restoring barrier functions. Interestingly, when compared to the H1L1I group, the mRNA expression levels of ZO-1 did not change in the H1L2I and H2L1I groups but significantly increased in the H2L1.5I group. Additionally, based on the cluster analysis heat map results and GO enrichment classifications, the H2L1.5I group contained several unique proteins with functional roles in membrane microdomain and membrane raft, indicating that simultaneously increasing leucine and isoleucine improved barrier functions in MAC-T cells better than leucine or isoleucine alone. However, the interactive effects of leucine and isoleucine on barrier functions require further investigation.

Interestingly, we found that leucine and isoleucine could act as antioxidants in MAC-T cells, which was expected. Additionally, we found that the protective mechanisms of leucine and isoleucine against oxidative stress were related to multiple regulatory pathways. Notably, the antagonism between leucine and isoleucine existed in antioxidative stress, but not in barrier functions or as anti-inflammatory activities. Based on this experimental data alone, the exact mechanism between the interaction of leucine and isoleucine was difficult to determine and remains unknown. Therefore, further research should be conducted to further analyze, expound, and prove this mechanism.

## 5. Conclusions

In conclusion, leucine was found to protect MAC-T cells from H_2_O_2_-induced oxidative stress by regulating the propanoate metabolism; valine, leucine and isoleucine degradation; and thermogenesis pathways, thereby generating more ATP to supplement energy demands. Moreover, the protective mechanisms of isoleucine against oxidative stress improved the deficit in peroxisome transport by regulating the peroxisome pathway and promoting acetyl-CoA production by regulating the propanoate metabolism pathway. The findings of this study enhance our understanding of the underlying protective mechanisms of leucine and isoleucine against oxidative damage.

## Figures and Tables

**Table 1:** Primer sequences used for real-time quantitative PCR (RT-qPCR) analysis.

Genes	Primer sequences (5′ to 3′)
Zonula occludens-1 (ZO-1)	F: 5′-GCGAAATGAGAAACAAGCACC-3′
	R: 5′-ATGAGTTGAGTTGGGCAGGAC-3′
Occludin	F: 5′-GCGAAATGAGAAACAAGCACC-3′
	R: 5′-ATGAGTTGAGTTGGGCAGGAC-3′
Claudin-1	F: 5′-AAGACGACGAGGCACAGAAGA-3′
	R: 5′-GAAGGTGCTGGCTTGGGATAG-3′
*β*-Actin	F: 5′-GCGTGGCTACAGCTTCACC-3′
	R: 5′-TTGATGTCACGGACGATTTC-3′

**Table 2:** DE proteins before and after leucine intervention in the propanoate metabolism, valine, leucine, and isoleucine degradation and thermogenesis pathways of MAC-T cells due to H_2_O_2_-induced oxidative damage.

Pathways	No.	Protein accession	Protein description	Gene name	H2L1I/H1L1I ratio	H2L1I/H1L1I *p* value
Propanoate metabolism	1	A0A3Q1LN22	Methylmalonate-semialdehyde dehydrogenase [acylating], mitochondrial	ALDH6A1	2.28	<0.001
	2	A7YWF1	Propionate-CoA ligase	ACSS2	1.61	0.004
Valine, leucine and isoleucine degradation	1	A0A3Q1LN22	Methylmalonate-semialdehyde dehydrogenase [acylating], mitochondrial	ALDH6A1	2.28	<0.001
	2	Q0NXR6	Isobutyryl-CoA dehydrogenase, mitochondrial	ACAD8	1.59	0.045
Thermogenesis	1	A0A3Q1N564	Protein arginine methyltransferase NDUFAF7	NDUFAF7	1.61	0.001
	2	A4FUH5	NADH dehydrogenase [ubiquinone] 1 alpha subcomplex assembly factor 4	NDUFAF4	1.53	0.003
	3	Q28851	ATP synthase subunit f, mitochondrial	ATP5MF	1.52	<0.001

**Table 3:** DE proteins before and after isoleucine intervention in the propanoate metabolism and peroxisome pathways of MAC-T cells due to H_2_O_2_-induced oxidative damage.

Pathways	No.	Protein accession	Protein description	Gene name	H1L2I/H1L1I ratio	H1L2I/H1L1I *p* value
Propanoate metabolism	1	A0A3Q1LN22	Methylmalonate-semialdehyde dehydrogenase [acylating], mitochondrial	ALDH6A1	1.95	0.001
	2	A7YWF1	Propionate-CoA ligase	ACSS2	1.51	0.017
Peroxisome	1	A0A3Q1LZN4	Peroxisomal membrane protein PEX16	PEX16	2.14	0.024
	2	O18778	Phytanoyl-CoA dioxygenase, peroxisomal	PHYH	1.70	0.034

## Data Availability

The original contributions presented in the study are included in the article material; further inquiries can be directed to the corresponding author.

## Supplementary Materials

Supplementary Materials Supplementary Table 1: MAC-T cells were firstly cultured in completed medium with or without 600 *μ*mol/L H_2_O_2_ for 6 h and then incubated in DMEM/F12 medium (1% antibiotics) with different combinations of leucine and isoleucine for 24 h. ^1^H corresponds to treatment of 600 *μ*mol/L H_2_O_2_ for 6 h; 1L and 2L correspond to 1 and 2 times the leucine concentration in DMEM/F12, which was 0.450 mmol/L; 1I, 1.5I, and 2I correspond to 1, 1.5, and 2 times the isoleucine concentration in DMEM/F12, which was 0.420 mmol/L. For example, H1L1I means that the MAC-T cells were firstly cultured in completed medium with 600 *μ*mol/L H_2_O_2_ for 6 h and then incubated in DMEM/F12 medium (1% antibiotics) with combinations of 0.450 mmol/L leucine and 0.420 mmol/L isoleucine for 24 h. Supplementary Table 2: effects of the different H_2_O_2_ concentrations for different times on the cellular viability (%) of MAC-T cells. ^1^Different letters (a–e) indicate significant differences at the same concentration (*p* < 0.05).

## References

[1] Drackley J. K. (1999). Biology of dairy cows during the transition period: the final frontier?. *Journal of Dairy Science*.

[2] Sordillo L. M., Aitken S. L. (2009). Impact of oxidative stress on the health and immune function of dairy cattle. *Veterinary Immunology and Immunopathology*.

[3] Pizzino G., Irrera N., Cucinotta M. (2017). Oxidative stress: harms and benefits for human health. *Oxidative Medicine and Cellular Longevity*.

[4] Gedik S., Erdemli M. E., Gul M. (2017). Hepatoprotective effects of crocin on biochemical and histopathological alterations following acrylamide-induced liver injury in Wistar rats. *Biomedicine & Pharmacotherapy*.

[5] Erdemli Z., Erdemli M. E., Gul M. (2021). Ameliorative effects of crocin on the inflammation and oxidative stress-induced kidney damages by experimental periodontitis in rat. *Iranian Journal of Basic Medical Sciences*.

[6] Forouharmehr A., Harkinezhad T., Qasemi-Panahi B. (2013). Effect of aflatoxin B1 on growth of bovine mammary epithelial cells in 3D and monolayer culture system. *Advanced Pharmaceutical Bulletin*.

[7] Huynh H. T., Robitaille G., Turner J. D. (1991). Establishment of bovine mammary epithelial cells (MAC-T): an in vitro model for bovine lactation. *Experimental Cell Research*.

[8] Wang T., Lee H. G., Zhen Y. G. (2018). Responses of MAC-T cells to inhibited stearoyl-CoA desaturase 1 during cis-9, trans-11 conjugated linoleic acid synthesis. *Lipids*.

[9] Wang T., Jeon S. W., Jung U. S., Kim M. J., Lee H. G. (2019). L-Lactate dehydrogenase B chain associated with milk protein content in dairy cows. *Animals*.

[10] Li T., Zhan Z. H., Lin Y. N. (2019). Biosynthesis of amino acids in Xanthomonas oryzae pv. oryzae is essential to its pathogenicity. *Microorganisms*.

[11] Sartori T., Antunes A. C. S., da Silva R. O. (2020). Branched chain amino acids improve mesenchymal stem cell proliferation, reducing nuclear factor kappa B expression and modulating some inflammatory properties. *Nutrition*.

[12] Hutson S. M., Sweatt A. J., Lanoue K. F. (2005). Branched-chain amino acid metabolism: implications for establishing safe intakes. *The Journal of Nutrition*.

[13] Bonvini A., Coqueiro A. Y., Tirapegui J., Calder P. C., Rogero M. M. (2018). Immunomodulatory role of branched-chain amino acids. *Nutrition Reviews*.

[14] Francesco B., Enzo N. (2017). Branched-chain amino acids differently modulate catabolic and anabolic states in mammals: a pharmacological point of view. *British Journal of Pharmacology*.

[15] Zheng S., Qin G. X., Zhen Y. G. (2021). Correlation of oxidative stress-related indicators with milk composition and metabolites in early lactating dairy cows. *Veterinary Medicine and Science*.

[16] Mao H., Wang C., Yu Z. (2019). Dietary leucine supplementation enhances the health of early weaned Hu lambs. *Animal Feed Science and Technology*.

[17] Yin L., Zhao Y., Zhou X. Q. (2020). Effect of dietary isoleucine on skin mucus barrier and epithelial physical barrier functions of hybrid bagrid catfish Pelteobagrus vachelli × Leiocassis longirostris. *Fish Physiology and Biochemistry*.

[18] Wang Q., Wang X. F., Xing T. (2020). The combined impact of xylo-oligosaccharides and gamma-irradiated Astragalus polysaccharides on growth performance and intestinal mucosal barrier function of broilers. *Poultry Science*.

[19] Kanehisa M., Goto S. (2000). KEGG: Kyoto encyclopedia of genes and genomes. *Nucleic Acids Research*.

[20] Wang B., Zhao L., Gao Z. D. (2021). Quantitative proteomic analysis of aberrant expressed lysine acetylation in gastrointestinal stromal tumors. *Clinical Proteomics*.

[21] Li L. Q., Lyu C. C., Li J. H. (2019). Physiological analysis and proteome quantification of alligator weed stems in response to potassium deficiency stress. *International Journal of Molecular Sciences*.

[22] Wu J. Q., Kosten T. R., Zhang X. Y. (2013). Free radicals, antioxidant defense systems, and schizophrenia. *Progress in Neuro-Psychopharmacology and Biological Psychiatry*.

[23] Hung W. C., Ling X. H., Chang C. C. (2017). Inhibitory effects of Siegesbeckia orientalis extracts on advanced glycation end product formation and key enzymes related to metabolic syndrome. *Molecules*.

[24] Zhao J., Liu Y., Jiang J. (2013). Effects of dietary isoleucine on the immune response, antioxidant status and gene expression in the head kidney of juvenile Jian carp (Cyprinus carpio var. Jian). *Fish & Shellfish Immunology*.

[25] Zhou C. P., Lin H. Z., Huang Z., Wang J., Wang Y., Yu W. (2020). Effects of dietary leucine levels on intestinal antioxidant status and immune response for juvenile golden pompano (*Trachinotus ovatus*) involved in Nrf2 and NF-*κ*B signaling pathway. *Fish & Shellfish Immunology*.

[26] Hu J., Nie Y. F., Chen S. F. (2017). Leucine reduces reactive oxygen species levels via an energy metabolism switch by activation of the mTOR-HIF-1*α* pathway in porcine intestinal epithelial cells. *International Journal of Biochemistry and Cell Biology*.

[27] Wu T., Zhang Y., Lv Y. (2018). Beneficial impact and molecular mechanism of Bacillus coagulans on piglets’ intestine. *International Journal of Molecular Sciences*.

[28] Shi J., Zhao Y., Wang Y. (2014). Inflammatory caspases are innate immune receptors for intracellular LPS. *Nature*.

[29] Jin X. L., Wang K., Liu H. Y., Hu F., Zhao F., Liu J. (2016). Protection of bovine mammary epithelial cells from hydrogen peroxide-induced oxidative cell damage by resveratrol. *Oxidative Medicine and Cellular Longevity*.

[30] Li H. D., Xu S. Z., Gao X., Ren H. Y. (2007). Structure of the bovine ACAD8 gene and the association of its polymorphism with the production traits. *Journal of Genetics and Genomics*.

[31] Lu J., Chen Z., Zhao H. (2020). ABAT and ALDH6A1, regulated by transcription factor HNF4A, suppress tumorigenic capability in clear cell renal cell carcinoma. *Journal of Translational Medicine*.

[32] Nie C. X., He T., Zhang W. J., Zhang G. L., Ma X. (2018). Branched chain amino acids: beyond nutrition metabolism. *International Journal of Molecular Sciences*.

[33] Han Y., Chen P., Zhang Y. Y. (2019). Synergy between auranofin and celecoxib against colon cancer in vitro and in vivo through a novel redox-mediated mechanism. *Cancers*.

[34] Hsu Y. J., Wang C. Y., Lee M. C., Huang C. C. (2020). Hepatoprotection by traditional essence of ginseng against carbon tetrachloride-induced liver damage. *Nutrients*.

[35] Kim P. K., Mullen R. T., Schumann U., Lippincott-Schwartz J. (2006). The origin and maintenance of mammalian peroxisomes involves a de novo PEX16-dependent pathway from the ER. *The Journal of Cell Biology*.

[36] Matsuzaki T., Fujiki Y. (2008). The peroxisomal membrane protein import receptor Pex 3p is directly transported to peroxisomes by a novel Pex 19p- and Pex 16p-dependent pathway. *The Journal of Cell Biology*.

[37] Li P., Yin Y. L., Li D. F., Kim S. W., Wu G. Y. (2007). Amino acids and immune function. *British Journal of Nutrition*.

[38] Benton D. A., Harper A. E., Spivey H. E., Elvehjem C. A. (1956). Leucine, isoleucine and valine relationships in the rat. *Archives of Biochemistry and Biophysics*.

[39] Osorio J. S., Trevisi E., Ji P. (2014). Biomarkers of inflammation, metabolism, and oxidative stress in blood, liver, and milk reveal a better immunometabolic status in peripartal cows supplemented with Smartamine M or Meta Smart. *Journal of Dairy Science*.

[40] Sun X. D., Jia H. D., Xu Q. S., Zhao C., Xu C. (2019). Lycopene alleviates H_2_O_2_-induced oxidative stress, inflammation and apoptosis in bovine mammary epithelial cells via the NFE2L2 signaling pathway. *Food & Function*.

[41] Mcfadden J. W. (2020). Review: Lipid biology in the periparturient dairy cow: contemporary perspectives. *Animal*.

[42] Lee J. H., Park E., Jin H. J. (2017). Anti-inflammatory and anti-genotoxic activity of branched chain amino acids (BCAA) in lipopolysaccharide (LPS) stimulated RAW 264.7 macrophages. *Food Science and Biotechnology*.

[43] Vijayan V., Pradhan P., Braud L. (2019). Human and murine macrophages exhibit differential metabolic responses to lipopolysaccharide - a divergent role for glycolysis. *Redox Biology*.

[44] Yang F., Wang A., Zeng X. F., Hou C., Liu H., Qiao S. (2015). Lactobacillus reuteri I5007 modulates tight junction protein expression in IPEC-J2 cells with LPS stimulation and in newborn piglets under normal conditions. *BMC Microbiology*.

[45] Barekatain R., Chrystal P. V., Howarth G. S., McLaughlan C. J., Gilani S., Nattrass G. S. (2019). Performance, intestinal permeability, and gene expression of selected tight junction proteins in broiler chickens fed reduced protein diets supplemented with arginine, glutamine, and glycine subjected to a leaky gut model. *Poultry Science*.

[46] Furuse M., Sasaki H., Fujimoto K., Tsukita S. (1998). A single gene product, claudin-1 or -2, reconstitutes tight junction strands and recruits occludin in fibroblasts. *The Journal of Cell Biology*.

[47] Wang Y., Tong J., Chang B., Wang B., Zhang D., Wang B. (2014). Effects of alcohol on intestinal epithelial barrier permeability and expression of tight junction-associated proteins. *Molecular Medicine Reports*.

