# Supplementary material for: Protective Mechanism of Leucine and Isoleucine against H2O2-Induced Oxidative Damage in Bovine Mammary Epithelial Cells
Source: Oxid Med Cell Longev. 2022 Mar 22;2022:4013575. doi: 10.1155/2022/4013575 (PMC8964234; doi:10.1155/2022/4013575)
Supplement: Supplementary Materials — Supplementary Table 1: MAC-T cells were firstly cultured in completed medium with or without 600 μmol/L H2O2 for 6 h and then incubated in DMEM/F12 medium (1% antibiotics) with different combinations of leucine and isoleucine for 24 h. 1H corresponds to treatment of 600 μmol/L H2O2 for 6 h; 1L and 2L correspond to 1 and 2 times the leucine concentration in DMEM/F12, which was 0.450 mmol/L; 1I, 1.5I, and 2I correspond to 1, 1.5, and 2 times the isoleucine concentration in DMEM/F12, which was 0.420 mmol/L. For example, H1L1I means that the MAC-T cells were firstly cultured in completed medium with 600 μmol/L H2O2 for 6 h and then incubated in DMEM/F12 medium (1% antibiotics) with combinations of 0.450 mmol/L leucine and 0.420 mmol/L isoleucine for 24 h. Supplementary Table 2: effects of the different H2O2 concentrations for different times on the cellular viability (%) of MAC-T cells. 1Different letters (a–e) indicate significant differences at the same concentration (p < 0.05). [file 4013575.f1.doc]

Supplementary Table 1: MAC-T cells were firstly cultured in completed medium with or without 600 μmol/L H_2_O_2_ for 6 h, then incubated in DMEM/F12 medium (1% antibiotics) with different combinations of leucine and isoleucine for 24 h.

| Treatments | H_2_O_2_, μmol/L, 6h  (in completed medium) | Combinations, mmol/L, 24 h  (in DMEM/F12 medium with 1% antibiotics) | |
| --- | --- | --- | --- |
|  |  | Leucine | Isoleucine |
| Control | 0 | 0.450 | 0.420 |
| H1L1I^1^ | 600 | 0.450 | 0.420 |
| H1L2I | 600 | 0.450 | 0.840 |
| H2L1I | 600 | 0.900 | 0.420 |
| H2L1.5I | 600 | 0.900 | 0.630 |

^1^ H correspond to treatment of 600 μmol/L H_2_O_2_ for 6 h; 1L and 2L correspond to 1 and 2 times the leucine concentration in DMEM/F12, which was 0.450 mmol/L; 1I, 1.5I and 2I correspond to 1, 1.5 and 2 times the isoleucine concentration in DMEM/F12, which was 0.420 mmol/L. For example, H1L1I means the MAC-T cells were firstly cultured in completed medium with 600 μmol/L H_2_O_2_ for 6 h, then incubated in DMEM/F12 medium (1% antibiotics) with combinations of 0.450 mmol/L leucine and 0.420 mmol/L isoleucine for 24 h.

Supplementary Table 2: Effects of the different H_2_O_2_ concentrations for different times on the cellular viability (%) of MAC-T cells.

| Treatments^1^ | 2 h | 4 h | 6 h | 8 h | 10 h |
| --- | --- | --- | --- | --- | --- |
| 0 μmol/L | 100.00 | 100.00 | 100.00 | 100.00 | 100.00 |
| 200 μmol/L | 97.11±1.91^a^ | 95.11±1.15^ab^ | 93.08±2.26^bc^ | 91.15±2.27^c^ | 86.02±2.02^d^ |
| 400 μmol/L | 93.03±2.59^a^ | 91.69±1.58^a^ | 88.16±1.57^b^ | 80.24±1.98^c^ | 70.22±0.98^d^ |
| 600 μmol/L | 89.41±0.88^a^ | 84.99±2.78^b^ | 75.91±1.45^c^ | 72.49±0.72^d^ | 61.06±0.13^e^ |
| 800 μmol/L | 82.91±1.92^a^ | 81.25±1.44^a^ | 64.58±2.11^b^ | 60.9±0.97^c^ | 54.57±0.40^d^ |
| 1000 μmol/L | 69.54±2.26^a^ | 68.51±0.42^a^ | 55.2±0.73^b^ | 52.88±0.73^c^ | 43.29±0.96^d^ |
| 1200 μmol/L | 13.57±0.66^a^ | 7.58±0.18^b^ | 3.35±0.58^c^ | 3.55±0.83^c^ | 2.53±0.40^d^ |

^1^ Different letters (a–e) indicate significant differences at the same concentration (*p* < 0.05).
